# Supplementary material for: Dietary and Sexual Correlates of Gut Microbiota in the Japanese Gecko, Gekko japonicus (Schlegel, 1836)
Source: Animals (Basel). 2023 Apr 16;13(8):1365. doi: 10.3390/ani13081365 (PMC10134999; doi:10.3390/ani13081365)
Supplement: Supplementary file 1 [file animals-13-01365-s001.zip › animals-2285250-supplementary.pdf]

animals

MDPI

Supplementary Figure and Tables

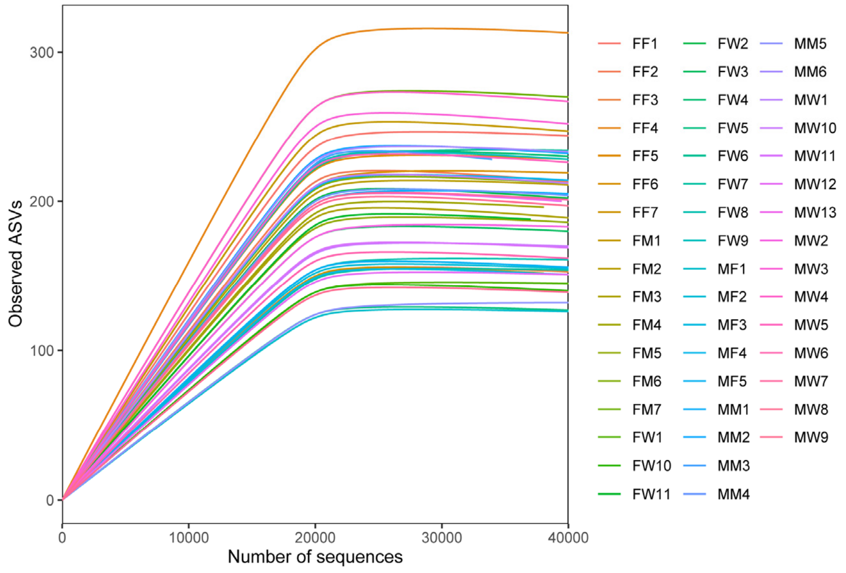

Figure S1. Rarefaction curves based on ASVs for individual fecal samples. Each color represents a sample.

Table S1. The number of valid reads and sequence information for each fecal sample.

Sample ID	Group	Raw reads	High-quality reads	Average sequence length	Minimum sequence length	Maximum sequence length	Accession number
FF1	FF	73684	40053	406.59	258	422	SAMC798099
FF2	FF	88949	60703	406.61	257	422	SAMC798104
FF3	FF	90123	51799	408.68	395	422	SAMC798108
FF4	FF	94885	66593	407.10	395	422	SAMC798112
FF5	FF	88354	44352	406.84	259	422	SAMC798130
FF6	FF	87440	53660	407.19	260	422	SAMC798131
FF7	FF	73619	38059	411.72	395	422	SAMC798132
FM1	FM	75766	41882	405.6	258	422	SAMC798096
FM2	FM	102760	70196	408.12	395	422	SAMC798098
FM3	FM	72769	40632	409.31	395	422	SAMC798101
FM4	FM	67705	38056	408.60	260	422	SAMC798103
FM5	FM	74409	43685	403.84	261	422	SAMC798105
FM6	FM	72310	43249	409.58	395	422	SAMC798107
FM7	FM	81193	59389	407.66	259	422	SAMC798110
FW1	FW	112652	66225	406.46	259	422	SAMC798095
FW2	FW	86098	43304	409.95	395	422	SAMC798097
FW3	FW	76989	41571	411.82	395	422	SAMC798100
FW4	FW	84867	46340	404.12	261	422	SAMC798102
FW5	FW	69268	42933	409.83	259	422	SAMC798106
FW6	FW	150444	86689	401.75	260	422	SAMC798109
FW7	FW	92941	52980	408.74	395	422	SAMC798111
FW8	FW	86275	48301	409.68	395	422	SAMC798137
FW9	FW	82425	50606	407.51	395	422	SAMC798138
FW10	FW	81409	47539	411.59	260	422	SAMC798139
FW11	FW	72672	37028	408.33	257	422	SAMC798140
MF1	MF	77935	47389	408.57	395	421	SAMC798115
MF2	MF	82673	45365	410.01	307	422	SAMC798122
MF3	MF	93453	52888	406.79	257	422	SAMC798123

Sample ID	Group	Raw reads	High-quality reads	Average sequence length	Minimum sequence length	Maximum sequence length	Accession number
MF4	MF	92104	41266	401.99	259	422	SAMC798124
MF5	MF	90387	50096	411.24	395	422	SAMC798129
MM1	MM	82136	45335	408.02	395	422	SAMC798114
MM2	MM	104528	55091	410.22	395	422	SAMC798117
MM3	MM	92997	33970	408.92	258	422	SAMC798119
MM4	MM	96831	44585	411.18	395	422	SAMC798121
MM5	MM	94575	57840	407.83	395	421	SAMC798126
MM6	MM	88806	51696	408.94	395	422	SAMC798128
MW1	MW	98013	54728	407.02	395	422	SAMC798113
MW2	MW	86628	56998	403.80	283	422	SAMC798116
MW3	MW	97957	70420	405.41	260	422	SAMC798118
MW4	MW	90098	60973	406.60	260	423	SAMC798120
MW5	MW	96383	51194	405.50	258	422	SAMC798125
MW6	MW	90030	47425	413.33	395	422	SAMC798127
MW7	MW	95586	53061	408.23	258	422	SAMC798133
MW8	MW	71594	47456	408.50	395	422	SAMC798134
MW9	MW	89601	45778	406.39	258	422	SAMC798135
MW10	MW	90782	53788	406.60	257	422	SAMC798136
MW11	MW	89660	55005	407.86	260	422	SAMC798141
MW12	MW	93227	55424	406.95	281	422	SAMC798142
MW13	MW	73681	39467	410.10	395	422	SAMC798143

Table S2. The number of bacterial amplicon sequence variants (ASVs) at different taxonomic levels in each fecal sample.

Sample ID	Group	ASVs	Genus	Family	Class	Order	Phylum
FF1	FF	211	68	46	14	29	8
FF2	FF	189	78	52	15	34	10
FF3	FF	146	43	34	13	25	8
FF4	FF	197	84	52	15	35	10
FF5	FF	211	59	35	12	21	9
FF6	FF	173	61	41	14	29	8
FF7	FF	132	41	31	12	19	8
FM1	FM	213	71	43	11	24	7
FM2	FM	189	75	51	14	32	10
FM3	FM	179	52	36	12	23	7
FM4	FM	188	60	40	12	27	8
FM5	FM	162	49	38	12	23	7
FM6	FM	178	72	45	12	27	8
FM7	FM	197	80	49	14	31	10
FW1	FW	126	55	34	10	21	7
FW2	FW	179	50	37	12	24	8
FW3	FW	167	51	37	10	21	7
FW4	FW	191	59	37	12	25	8
FW5	FW	115	41	29	10	19	7
FW6	FW	206	56	38	13	26	8
FW7	FW	145	42	28	10	19	7
FW8	FW	147	41	28	10	18	7
FW9	FW	173	56	40	11	23	6
FW10	FW	114	35	32	12	22	7
FW11	FW	171	48	31	12	21	9
MF1	MF	122	35	30	12	22	8
MF2	MF	150	43	32	13	21	8
MF3	MF	142	45	33	12	22	8

Sample ID	Group	ASVs	Genus	Family	Class	Order	Phylum
MF4	MF	187	53	34	12	22	7
MF5	MF	149	43	32	11	19	8
MM1	MM	197	58	38	11	22	8
MM2	MM	212	56	37	11	22	6
MM3	MM	214	60	39	13	25	9
MM4	MM	186	46	31	13	21	8
MM5	MM	114	34	33	11	23	6
MM6	MM	210	58	44	12	27	7
MW1	MW	204	54	33	13	23	8
MW2	MW	134	52	40	11	28	7
MW3	MW	128	59	44	14	28	9
MW4	MW	183	77	47	13	30	9
MW5	MW	204	50	33	11	20	7
MW6	MW	147	36	24	11	16	7
MW7	MW	190	48	29	12	19	8
MW8	MW	117	39	28	12	19	8
MW9	MW	172	53	35	14	23	9
MW10	MW	161	48	31	12	20	8
MW11	MW	152	58	40	14	27	9
MW12	MW	135	44	26	10	18	7
MW13	MW	173	50	35	12	22	7

Table S3. The relative abundance of unique bacterial taxon among different groups based on the Kruskal–Wallis H test. The letters "f" and "g" indicate family and genus, respectively.

Taxonomy	*df*	*H*	*p*
f__Caulobacteraceae	5	11.76	0.04
f__ Desulfovibrionaceae	5	13.85	0.02
f__Eggerthellaceae	5	17.08	0.004
f__Erysipelatoclostridiaceae	5	11.23	0.05
f__Marinifilaceae	5	13.23	0.02
f__Tannerellaceae	5	10.51	0.06
g__*Bacteroides*	5	17.77	0.003
g__*Clostridium_sensu_stricto_1*	5	14.60	0.01
g__*Desulfovibrio*	5	16.18	0.006
g__*Eggerthella*	5	15.56	0.008
g__*Odoribacter*	5	13.62	0.02

Table S4. The relative abundance of unique predicated functions among different groups based on the Kruskal-Wallis H test.

Level name	*df*	*H*	*p*
Metabolism丨Energy metabolism	5	15.66	0.008
Environmental Information Processing	5	13.22	0.02
Environmental Information Processing丨Membrane transport	5	13.54	0.02
Metabolism丨Biosynthesis of other secondary metabolites	5	12.35	0.03
Metabolism丨Metabolism of cofactors and vitamins	5	12.03	0.03
ko00010	5	11.47	0.04
ko00051	5	11.26	0.05
ko00340	5	12.82	0.02
ko00473	5	11.90	0.04
ko00720	5	14.23	0.01
ko00790	5	12.88	0.02
ko01055	5	13.40	0.02
